# Vincristine enhances amoeboid-like motility via GEF-H1/RhoA/ROCK/Myosin light chain signaling in MKN45 cells

**DOI:** 10.1186/1471-2407-12-469

**Published:** 2012-10-12

**Authors:** Masato Eitaki, Tohru Yamamori, Shunsuke Meike, Hironobu Yasui, Osamu Inanami

**Affiliations:** 1 Department of Environmental Veterinary Sciences, Laboratory of Radiation Biology, Graduate School of Veterinary Medicine, Hokkaido University, Sapporo, 060-0818, Japan

## Abstract

**Background:**

Anti-cancer drugs are widely used in cancer treatment frequently combined with surgical therapy and/or radiation therapy. Although surgery and radiation have been suggested to facilitate invasion and metastasis of tumor cells in some cases, there is so far little information about the effect of anti-cancer drugs on cellular invasive ability and metastasis. In this study, using four different anti-cancer drugs (vincristine, paclitaxel, cisplatin and etoposide), we examined whether these drugs influence the invasive ability of tumor cells.

**Methods:**

Human gastric adenocarcinoma MKN45 cells were used to evaluate the effect of anti-cancer drugs. After drug treatment, cellular invasive ability was assessed using the Matrigel invasion chamber. Cytoskeletal changes after treatment were examined microscopically with F-actin staining. In addition, we monitored cellular motility in 3D matrigel environment by time-lapse microscopic analysis. The drug-induced activation of RhoA and ROCK was evaluated by pull-down assay and Western blotting using an antibody against phosphorylated myosin light chain (MLC), respectively. Where necessary, a ROCK inhibitor Y27632 and siRNA for guanine nucleotide exchange factor-H1 (GEF-H1) were applied.

**Results:**

Among all drugs tested, only vincristine stimulated the invasive ability of MKN45 cells. Microscopic analysis revealed that vincristine induced the formation of non-apoptotic membrane blebs and amoeboid-like motility. Vincristine significantly enhanced RhoA activity and MLC phosphorylation, suggesting the involvement of RhoA/ROCK pathway in the vincristine-induced cytoskeletal reorganization and cellular invasion. Furthermore, we found that Y27632 as well as the siRNA for GEF-H1, a RhoA-specific activator, attenuated MLC phosphorylation, the formation of membrane blebs and the invasive ability after vincristine treatment.

**Conclusions:**

These results indicate that vincristine activates GEF-H1/RhoA/ROCK/MLC signaling, thereby promoting amoeboid-like motility and the invasive ability of MKN45 cells.

## Background

Metastasis is one of the most fatal aspects of cancer. In order to improve the status of cancer patients, consideration for metastasis and invasion is necessary. In general, cancer treatment is carried out by single or combined therapy of anti-cancer drugs, surgery and ionizing radiation. However, surgery and radiotherapy have been reported to have a risk of undesirable metastasis or invasion [1-4]. For example, Zhai et al. have suggested that radiation enhances the invasiveness of glioblastoma cells [5]. In addition to the risk of surgery- and radiation-induced tumor metastasis, an anti-cancer drug doxorubicin, which intercalates into DNA and inhibits DNA topoisomerase II, has been reported to stimulate metastasis and invasion of tumor cells via transforming growth factor-β (TGF-β) signaling in breast cancer cells [3,6]. Because anti-cancer drugs influence various signal transduction pathways other than those associated with tumor growth and cell death, it might be possible that they enhance metastasis or invasion as their side effects.

Currently, many anti-cancer drugs are available and they have a variety of action mechanisms. These include microtubule perturbation by vincristine and paclitaxel, DNA crosslinking by cisplatin, and the inhibition of DNA topoisomerase by etoposide. Although the action mechanisms of anti-cancer drugs are distinct depending on the drugs, there are studies reporting the various types of anti-cancer drugs to influence tumor cell motility and metastasis. For example, microtubule agonists such as paclitaxel and vincristine have been shown to affect cellular motility [7-11]. Vinca alkaloids including vincristine were shown to inhibit directional migration via the abolishment of the cytoplasmic microtubule complex in mouse fibrosarcoma MO_4_ cells [8]. Paclitaxel was reported to decrease invasion and metastasis via the inhibition of extracellular matrix degrading factors in human prostatic PC-3 ML cells and human ovarian Ovcar-3 cells [10,11]. In addition, Mashino et al. have demonstrated that etoposide inhibits cellular invasion by the induction of a metastasis suppresser gene KAI1 in several cells including human lung adenocarcinoma A549 cells [12].

Each cancer is unique and heterogeneous, and different types of cancer respond differently to therapeutic modalities. For some cancers, survival rates after radiotherapy are high (for example, early stage larynx cancer and non-small-cell lung cancer), whereas for many other cancers they are not (for example, glioblastoms and sarcomas) [13]. For chemotherapy, because some cancers are susceptible to specific types of anti-cancer drugs while others are not, they are prescribed depending on their efficacy to the types of the cancer to be treated. For example, it has been reported that breast cancer responds well to 5-fluorouracil while cholangiocarcinoma doesn’t [14,15]. Among all human cancers, gastric cancer is the second frequent type of cancer in the world, and the rate of incidence varies with area, especially high in Asia, South America and Eastern Europe [16]. Although locally circumscribed tumors can be treated with surgery, radiation and/or endoscopic procedures, majority of patients develop metastases at some point during the course of their illness [16]. Metastatic tumors can be treated only with systemic chemotherapy, but some chemotherapeutic drugs such as doxorubicin promote metastasis in certain conditions as described above [3,6]. Nevertheless, the effect of anti-cancer drugs on the metastatic activity of gastric cancer cells has been hardly investigated. In this study, to determine the relationship between anti-cancer drugs and the metastatic activity of tumor cells, we examined whether four different types of anti-cancer drugs (vincristine, paclitaxel, cisplatin and etoposide) could influence the invasive ability of gastric adenosarcoma MKN45 cells.

## Methods

### Reagents

Vincristine, paclitaxel, cisplatin, etoposide and Y27632 were purchased from Wako Pure Chemical Co. (Osaka, Japan). Alexa Fluor 488 phalloidin and TRITC phalloidin were purchased from Invitrogen (Carlsbad, CA) and Sigma-Aldritch (St. Louis, MO), respectively. WST-1 and 1-methoxy PMS were purchased from Dojindo (Kumamoto, Japan).

### Antibodies

The following antibodies were used: cleaved caspase-3 (#9661; Cell Signaling Technology, Beverly, MA), RhoA (ARH03; Cytoskeleton, Denver, CO), MLC (#3672; Cell Signaling Technology), phosphorylated MLC (pMLC) (#3671; Cell Signaling Technology), GEF-H1 (#4076; Cell Signaling Technology) and actin (sc-1615; Santa Cruz Biotechnology, Santa Cruz, CA).

### Cell culture

Human gastric adenocarcinoma MKN45 cells were maintained in RPMI1640 medium (Invitrogen) containing 10% fetal bovine serum (FBS) (RPMI1640/10% FBS) at 37°C in a humidified atmosphere of 5% CO_2_.

### Invasion assay

Cellular invasive ability was evaluated using a BD BioCoat™ Matrigel™ Invasion Chamber (8 μm pore size, 24-well plate, BD Biosciences, Billerica, MA). Cells (1 × 10^6^) were suspended in 500 μl of serum-free RPMI1640 with or without drugs, and they were loaded into the upper chamber, followed by the addition of 750 μl of RPMI1640/10% FBS into the lower chamber. After incubation for 24 h at 37°C in a humidified atmosphere containing 5% CO_2_, non-invading cells were removed from the upper side of the membrane using cotton tips. Invading cells on the lower side of the membrane were fixed with 100% methanol, and stained with 1% toluidine blue and 1% sodium borate. All the invading cells were counted using a light microscope.

### WST-1 assay

Cells (1 × 10^4^ per well) were seeded into each well of a 96-well plate in 100 μl of RPMI1640/10% FBS with or without drugs. After incubation for 24 h at 37°C in a 5% CO_2_ humidified atmosphere, 10 μl of WST-1 solution (3.24 μg/μl WST-1, 70 ng/μl 1-Methoxy PMS, and 20 mM HEPES-NaOH [pH 7.4]) was added to each well. The cells were incubated for 1 h at 37°C in a humidified atmosphere containing 5% CO_2_, and the absorbance of each well was recorded at 450 nm using a Model 680 Microplate Reader (Bio-Rad Laboratories, Hercules, CA). Cell viability was expressed as a percentage relative to the absorbance obtained from the well containing non-treated control cells.

### F-actin staining and microscopic analysis

Cells (2.5 × 10^5^ per well) were suspended in 2 ml of serum-free RPMI1640 with or without drugs, and seeded on coverslips coated with 1% gelatin. After incubation for 24 h at 37°C in a humidified atmosphere containing 5% CO_2_, cells on the coverslips were fixed with 3.7% paraformaldehyde for 15 min at room temperature, and permeabilized with 0.2% Triton X-100 for 15 min, followed by blocking with 1% bovine serum albumin (BSA) for 30 min. The specimens were incubated with 2.5% Alexa Fluor 488 phalloidin containing 1% BSA and 0.1% Triton X-100 for 20 min, followed by staining with 300 nM DAPI for 5 min. After mounting, fluorescence imaging was performed with an LSM 700 confocal microscope (Carl Zeiss, Oberkochen, Germany) controlled by the Zeiss Efficient Navigation software with a ×100 objective lens.

### Detection of apoptotic cells with membrane blebs

Cells were seeded on gelatin-coated coverslips and incubated overnight. The cells were treated with vehicle, 15 μM vincristine or 100 μM etoposide. After treatment, they were fixed with 3.7% paraformaldehyde and stained with an anti-cleaved caspase-3 antibody, TRITC phalloidin and DAPI.

### Time-lapse imaging

Cells (1.25 × 10^4^) were suspended in 25 μl of serum-free RPMI1640 with or without 30 μM vincristine, and mixed with 25 μl of Matrigel (BD Biosciences). The cell suspension (25 μl) was dropped on a 35 mm glass-bottom dish using a chilled pipette tip, and was spread flat by putting a 15 mm glass coverslip on it. After gelating the Matrigel by incubating for 5 min at 37°C in a 5% CO_2_ humidified atmosphere, 2 ml of RPMI1640/10% FBS was added to the dish. The cells moving in the Matrigel were monitored at 37°C in a humidified atmosphere containing 5% CO_2_ using an ECLIPSE TE2000-E microscope (Nikon, Tokyo, Japan) with a ×100 objective lens and a RETIGA EXi FAST 1394 CCD digital camera (Qimaging, Burnaby, Canada). Differential interference contrast (DIC) images were acquired every minute for 1 h. Time-lapse movies (1 h per 9.15 sec) were created using an Windows Movie Maker software.

### RhoA activity assay

RhoA activity was evaluated using a RhoA activation assay kit (BK036; Cytoskeleton) according to the manufacturer's instructions. After starvation for 24 h in serum-free RPMI1640, cells were treated with or without vincristine up to 60 min at 37°C in a humidified atmosphere containing 5% CO_2_. The cells were then rinsed with ice-cold PBS and suspended in 400 μl of cell lysis buffer A (50 mM Tris–HCl [pH 7.5], 10 mM MgCl_2_, 0.5 M NaCl, 2% Igepal CA-630, 1 mM PMSF, 10 μg/ml leupeptin, 10 μg/ml aprotinin and 10 μg/ml pepstatin). Cell lysates were centrifuged for 2 min at 10,000 g, and supernatants were collected. Rhotekin beads (33.3 μg) were added to the cell extracts (2 mg protein in 1 ml of cell lysis buffer A) and they were rotated for 1 h at 4°C. After washing the beads with wash buffer (25 mM Tris–HCl [pH 7.5], 30 mM MgCl_2_, and 40 mM NaCl), proteins were released from the beads by boiling for 2 min in 15 μl of 2× Laemmli sample buffer (0.125 M Tris–HCl [pH 6.8], 10% β-mercaptoethanol, 4% SDS, 20% glycerol, and 0.004% bromophenol blue). The proteins were separated by SDS-PAGE, transferred to membranes, and analyzed by Western blotting using an anti-RhoA antibody (1:500 dilution) for active RhoA. The remaining extracts (50 μg) were also analyzed by Western blotting with the anti-RhoA antibody for total RhoA.

### MLC phosphorylation

After starvation for 24 h in serum-free RPMI1640, cells were treated with or without vincristine up to 60 min at 37°C in a humidified atmosphere containing 5% CO_2_. The cells were then rinsed with ice-cold PBS and suspended in 100 μl of cell lysis buffer B (30 mM HEPES-NaOH [pH 7.4], 100 mM NaCl, 20 mM NaF, 40 mM Na_4_P_2_O_7_, 1 mM Na_3_VO_4_, 1 mM EGTA, 1% Triton X-100, 1 mM PMSF, 10 μg/ml leupeptin, 10 μg/ml aprotinin and 10 μg/ml pepstatin). Cell lysates were centrifuged for 15 min at 20,000 g, and supernatants were collected. Extracts (80 μg) were separated by SDS-PAGE, transferred to membranes, and analyzed by Western blotting using an anti-MLC antibody (1:1000 dilution) or anti-pMLC antibody (1:1000 dilution).

### RNA interference

GEF-H1 Stealth Select RNAi™ siRNA (HSS113587, Invitrogen) and Stealth RNAi™ Negative Control Medium GC Duplex (12935–300, Invitrogen) were used. Cells were transfected with these siRNAs (200 pmol per 35 mm dish or 400 pmol per 60 mm dish) using Lipofectamine 2000 (Invitrogen). At 24 h after transfection, the culture medium was replaced with fresh RPMI1640/10% FBS.

To check the GEF-H1 expression level, transfected cells were rinsed with ice-cold PBS and suspended in cell lysis buffer B. Lysates were centrifuged for 15 min at 20,000 g, and supernatants were collected. Extracts (60 μg) were separated by SDS-PAGE, transferred to membranes, and analyzed by Western blotting using an anti-GEF-H1 antibody (1:1000 dilution).

### Statistical analysis

Values are presented as means ± S.E. of at least three independent experiments. Statistical significance was determined by Student’s *t* test, Welch’s *t* test or paired *t* test depending on the situation. *P* < 0.05 was considered significant.

## Results

### Vincristine enhances invasive ability

**Figure 1:**
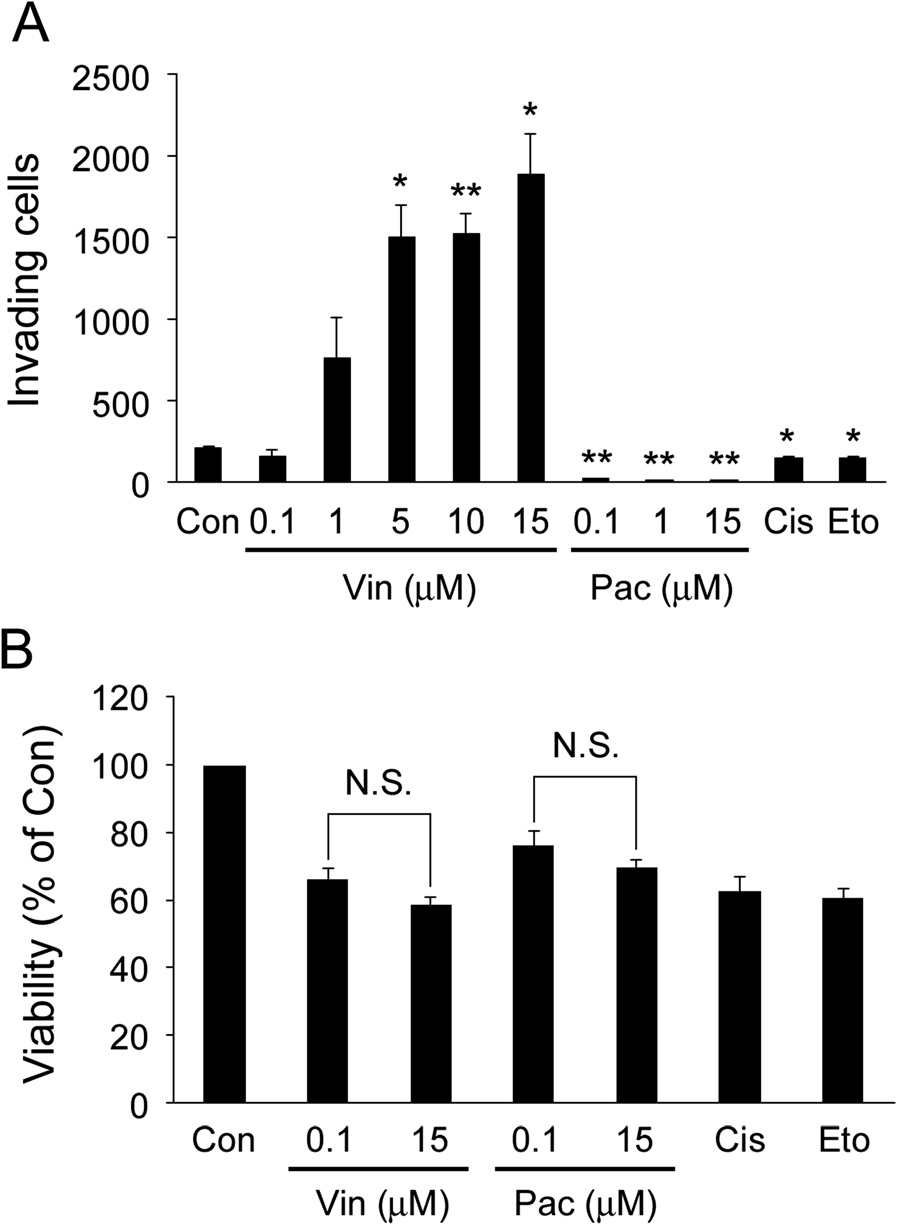
**Invasive ability enhanced by vincristine.A**. The number of invading cells measured by the invasion assay. Cells (1 × 10^6^) were seeded into the upper chamber in the presence or absence of anti-cancer drugs. After 24 h incubation, the invading cells were fixed, and stained by toluidine blue. Total numbers of the stained cells were counted using a microscope. Con, non-treated control; Vin, vincristine; Pac, paclitaxel; Cis, cisplatin (15 μM); Eto, etoposide (20 μM). The graph shows mean ± S.E. of three independent experiments. *, *P* < 0.05; **, *P* < 0.01 versus Con. **B**. Cells (1 × 10^4^) were seeded into wells of a 96-well plate in the presence or absence of anti-cancer drugs. After 24 h incubation and the following 1 h incubation with WST-1 solution, the absorbance at 450 nm was recorded using a microplate reader. Cell viability is expressed as percentages relative to the viability obtained for non-treated control. Con, non-treated control; Vin, vincristine; Pac, paclitaxel; Cis, cisplatin (15 μM); Eto, etoposide (20 μM). The graph shows mean ± S.E. of three independent experiments. N.S., not-significant.

To examine whether four different anti-cancer drugs (vincristine, paclitaxel, cisplatin and etoposide) affected cellular invasive ability, we performed invasion assay. After MKN45 cells were treated with each anti-cancer drug, the cellular invasive ability was analyzed using the Matrigel invasion chamber. As shown in Figure 1A, vincristine enhanced cellular invasive ability in a concentration-dependent manner whereas paclitaxel completely blocked it. Cisplatin and etoposide also inhibited it significantly, but that was not as much as paclitaxel did. Next, cell viability was evaluated by WST-1 assay to assess whether it was affected by the treatment with the drugs. Vincristine, cisplatin and etoposide decreased the viability of MKN45 cells about 35% compared to the non-treated control, whereas paclitaxel decreased it about 30% (Figure 1B). There was no significant difference in the cell viability between 0.1 and 15 μM vincristine treatment. Likewise, there was no significant difference in the cell viability between 0.1 and 15 μM paclitaxel treatment. These results indicated that vincristine enhanced cellular invasive ability independently of the effect on cell viability, and that microtubule depolymerization by vincristine, neither microtubule polymerization by paclitaxel nor DNA damage by cisplatin and etoposide, elevated the invasive ability of MKN45 cells.

### High concentration vincristine induces amoeboid-like motility

**Figure 2:**
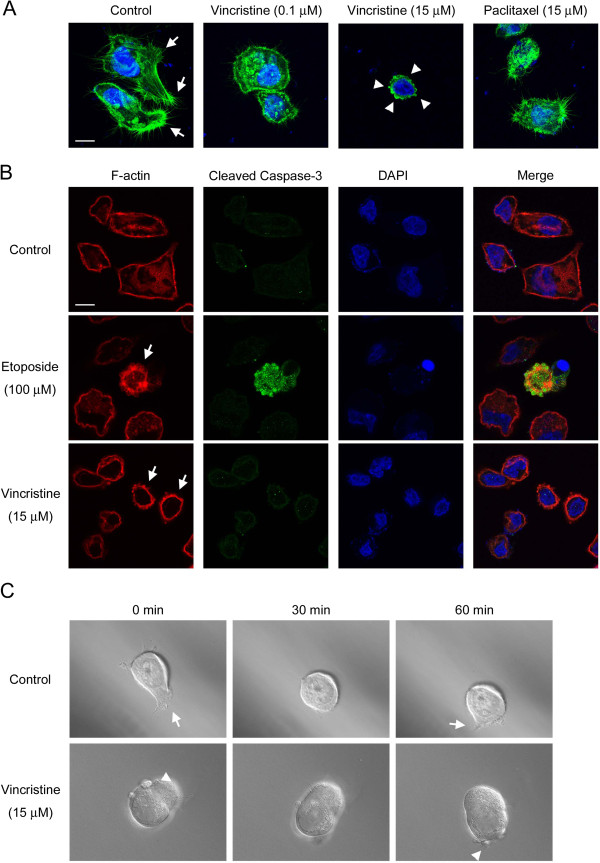
**Amoeboid**-**like motility observed after vincristine treatment. A**. Cellular morphology after 24 h drug treatment. Cells (2.5 × 10^5^) were seeded into wells of 6-well plates and incubated for 24 h on gelatin-coated coverslips with or without vincristine or paclitaxel. F-actin was labeled with Alexa Fluor 488 phalloidin, and nuclei were labeled with DAPI. F-actin (green) and nuclei (blue) were analyzed using confocal microscopy. Arrows indicate cell protrusions. Arrowheads indicate membrane blebs. White bars, 10 μm. **B**. Detection of blebbing cells and apoptosis. Cells on gelatin-coated coverslips were treated with vehicle or drugs, fixed and stained with TRITC phalloidin, cleaved caspase-3 antibody and DAPI. F-actin (red), cleaved caspase-3 (green) and DAPI (blue) were analyzed using confocal microscopy. Arrows indicate the cells with membrane blebs. White bars, 10 μm. **C**. DIC images from supplemental videos of the control cell (Additional file 1: Video S1) and the 15 μM vincristine-treated cell (Additional file 2: Video S2). Cells (6.25 × 10^3^) were confined to a 50% Matrigel environment with or without 15 μM vincristine on a 35 mm glass-bottom dish by a coverslip. Frames show the cells at the indicated times. Arrows indicate cell protrusions. Arrowheads indicate membrane blebs.

To understand how vincristine enhanced the invasive ability of MKN45 cells, we examined the morphology and motility of the vincristine-treated cells. Recently, it has been demonstrated that cancer cells display two different modes of cell motility, namely, mesenchymal motility and amoeboid-like motility, in an *in vitro* 3D environment as well as *in vivo*[17-19]. Mesenchymal motility is characterized by the elongated cellular morphology with cell protrusions, the integrin-dependent adhesion to extracellular matrix (ECM), and the degradation of the ECM by matrix metalloproteinases (MMPs) [20-23]. On the other hand, amoeboid-like motility is characterized by the rounded cellular morphology with membrane blebs, less adhesiveness to ECM, and the MMP-independent invasion through gaps of the ECM by squeezing [24-27]. To analyze the effect of vincristine on the cytoskeleton, F-actin was labeled with Alexa Fluor 488 phalloidin after vincristine treatment. As shown in Figure 2A, non-treated control cells formed numerous cell protrusions, which are often associated with mesenchymal motility. While 0.1 μM vincristine and 15 μM paclitaxel caused the reduction of cell protrusions, 15 μM vincristine induced the formation of membrane blebs, a hallmark of amoeboid-like motility.

Because membrane blebbing is frequently associated with apoptosis, we examined whether the vincristine-induced blebbing cells were apoptotic. The cells after drug treatment were co-stained with phalloidin and cleaved caspase-3 antibody to detect blebbing cells and apoptotic cells, respectively. We observed that etoposide increased the cleaved caspase-3-positive cells, indicating the induction of apoptosis by etoposide treatment (Figure 2B). As expected, these cleaved caspase-3-positive cells had membrane blebs. On the other hand, vincristine treatment stimulated the formation of membrane blebs, but the blebbing cells were not cleaved caspase-3-positive. Therefore, vincristine-induced membrane blebs were suggested to be independent of apoptosis.

We next investigated the cellular motility in a 3D Matrigel environment using the time-lapse imaging technique to monitor the mode of cellular motility directly. We observed that non-treated cells had the mesenchymal motility accompanied by the cell protrusions (Figure 2C and Additional file 1: Video S1). On the other hand, cells treated with 15 μM vincristine showed the amoeboid-like motility accompanied by the membrane blebs (Figure 2C and Additional file 2: Video S2 and Additional file 3: Video S3). These results indicated that, at high concentration, vincristine induced amoeboid-like motility in MKN45 cells.

### High concentration vincristine activates RhoA

**Figure 3:**
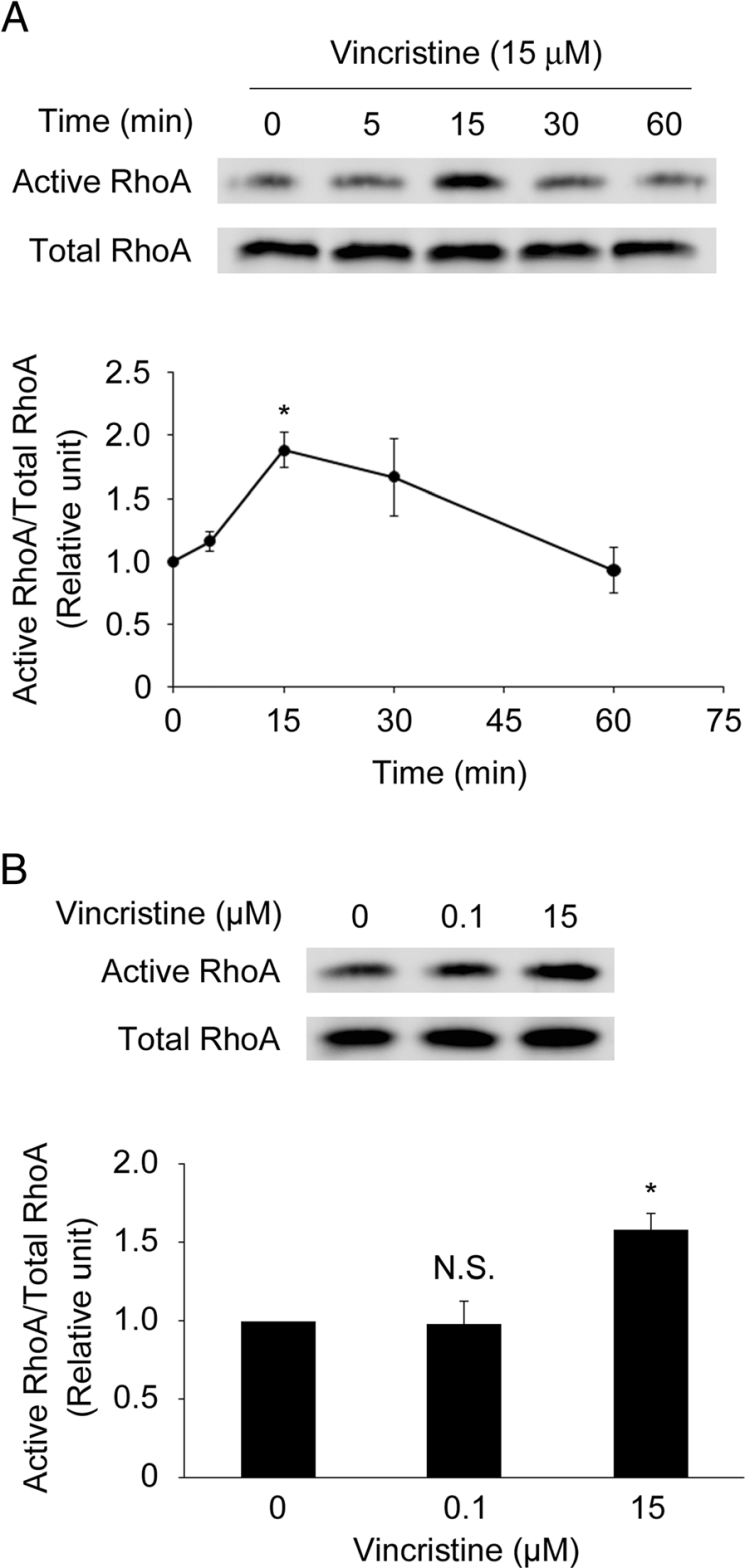
**RhoA activated by vincristine. A** and **B**. Cells were starved in serum-free RPMI1640 for 24 h, and then exposed to vincristine. After vincristine treatment, the cells were harvested to evaluate RhoA activity by rhotekin-based pull-down assay. RhoA in pull-down samples (active RhoA) and in total lysates (total RhoA) were detected by Western blotting using an anti-RhoA antibody. **A**, time-course; **B**, concentration-response at 15 min. The blots in (**A**) and (**B**) are representative of three independent experiments. The blots were quantified by densitometry, and the results were expressed as ratio relative to the values obtained in non-treated control cells (0 min or 0 μM). The graphs in (**A**) and (**B**) show means ± S.E. of three independent experiments. *, *P* < 0.05 versus control.

These results prompted us to determine how vincristine induced the formation of membrane blebs and amoeboid-like motility. Rho GTPases have been known to be essential for cellular motility [28-30]. Recent reports suggest that RhoA, one of the Rho GTPases, plays a crucial role in these events through the activation of the downstream targets such as ROCK and MLC [31-35]. To test whether vincristine affects RhoA activity in MKN45 cells, we assessed it by pull-down assay. RhoA activity increased transiently after 15 μM vincristine treatment, peaking at 15 min after the treatment, followed by a decrease to the basal level (Figure 3A). Whereas 15 μM vincristine significantly increased RhoA activity, 0.1 μM vincristine did not affect it (Figure 3B). These results indicated that 15 μM vincristine stimulated RhoA activity.

### High concentration vincristine promotes MLC phosphorylation

**Figure 4:**
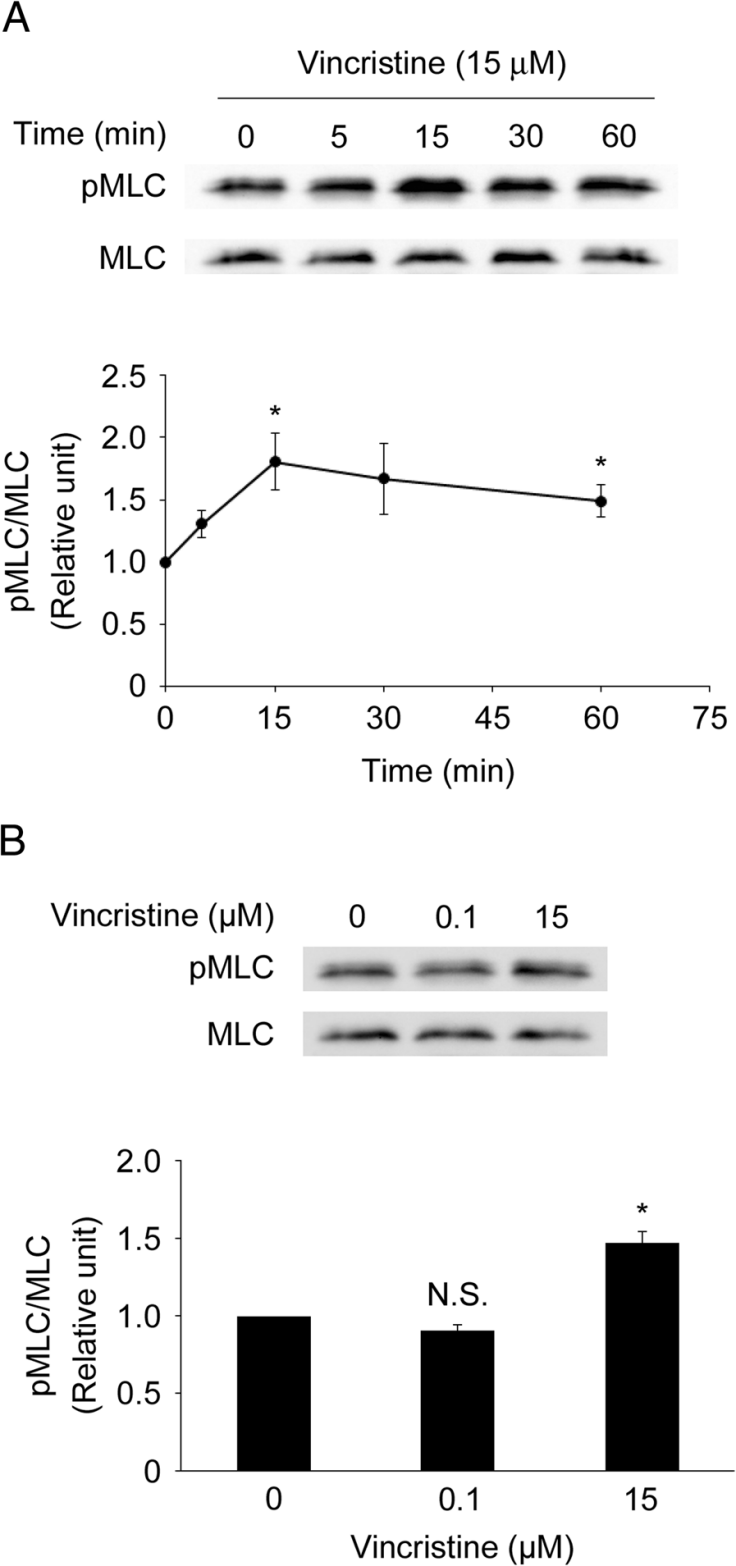
**MLC phosphorylation promoted by vincristine. A** and **B**. Cells were starved in serum-free RPMI1640 for 24 h, and then treated with or without vincristine. After vincristine treatment, the cells were harvested to evaluate MLC phosphorylation by Western blotting. **A**, time-course; **B**, concentration-response at 15 min. The blots in (**A**) and (**B**) are representative of three independent experiments. The blots were quantified by densitometry, and the results were expressed as a ratio relative to the values obtained in non-treated control cells (0 min or 0 μM). The graphs in (**A**) and (**B**) show means ± S.E. of three independent experiments. *, *P* < 0.05 versus control.

Because it is reported that RhoA activation leads to the phosphorylation of MLC via ROCK [33], we assessed MLC phosphorylation in vincristine-treated cells by Western blotting. MLC phosphorylation was increased transiently after 15 μM vincristine treatment, peaking at 15 min, followed by a gradual decrease up to 60 min (Figure 4A). MLC phosphorylation in cells treated with 15 μM vincristine was significantly higher than in non-treated control cells (Figure 4B). However, 0.1 μM vincristine treatment did not affect MLC phosphorylation. These results indicated that 15 μM vincristine promoted MLC phosphorylation.

### ROCK mediates vincristine-induced MLC phosphorylation, membrane blebbing and invasive ability

**Figure 5:**
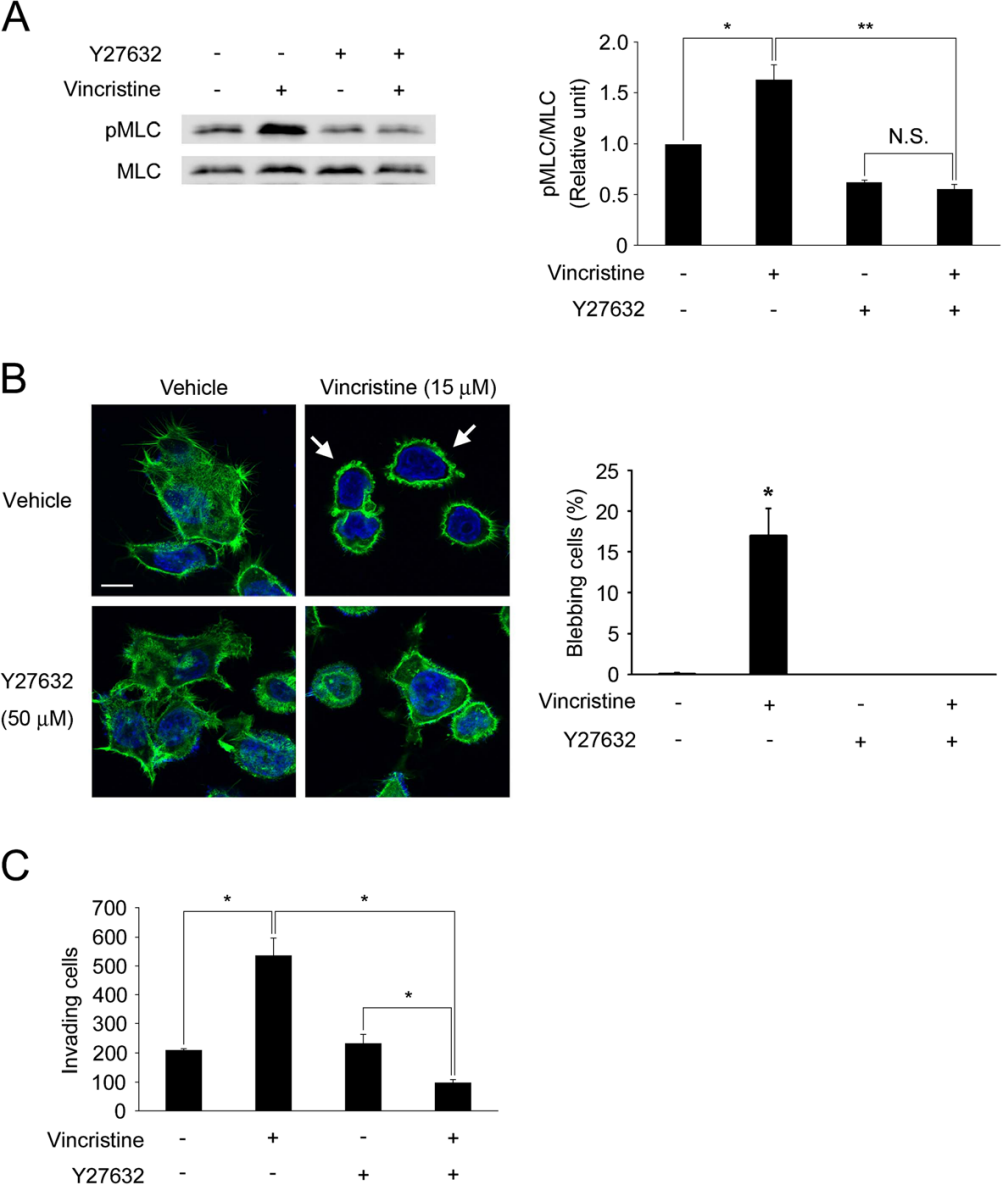
**Involvement of ROCK in vincristine-enhanced invasive ability. A**. Cells were starved in serum-free RPMI1640 for 24 h, and then treated with or without 15 μM vincristine and/or 50 μM Y27632 for 15 min. The cells were then harvested to evaluate MLC phosphorylation by Western blotting using anti-MLC and anti-pMLC antibodies. The blots in (**A**) are representative of four independent experiments. The blots were quantified by densitometry, and the results were expressed as ratio relative to the values obtained in non-treated control cells. The graph in (**A**) shows mean ± S.E. of four independent experiments. *, *P* < 0.05; **, *P* < 0.01. **B**. Cells on gelatin-coated coverslips were treated with drugs, fixed and stained with Alexa Fluor 488 phalloidin and DAPI. F-actin (green) and nuclei (blue) were analyzed using confocal microscopy. Arrows indicate the cells with membrane blebs. White bars, 10 μm. The graph shows means ± S.E. of three independent experiments. *, *P* < 0.05. **C**. Cells (1 × 10^6^) were seeded into the upper chamber with or without 15 μM vincristine and/or 50 μM Y27632. After 24 h incubation, the invading cells were fixed and stained with toluidine blue. Total numbers of the stained cells were counted using a microscope. The graphs show means ± S.E. of three independent experiments. *, *P* < 0.05.

To determine whether ROCK mediated vincristine-induced MLC phosphorylation, we performed Western blotting using a ROCK inhibitor Y27632. Fifteen micromolar vincristine significantly increased MLC phosphorylation, and this increase was clearly diminished by 50 μM Y27632 (Figure 5A). When the effect of Y27632 on the vincristine-induced membrane blebbing was evaluated, it abolished the formation of membrane blebs by vincristine (Figure 5B). In addition, we determined whether Y27632 affected the vincristine-induced cellular invasive ability. While Y27632 did not affect the basal invasive ability of MKN45 cells, it significantly inhibited the invasive ability in cells treated with 15 μM vincristine (Figure 5C). These results indicated that vincristine enhanced the membrane blebbing and the cellular invasive ability via ROCK-mediated MLC phosphorylation.

### GEF-H1 mediates vincristine-induced MLC phosphorylation, membrane blebbing and invasive ability

**Figure 6:**
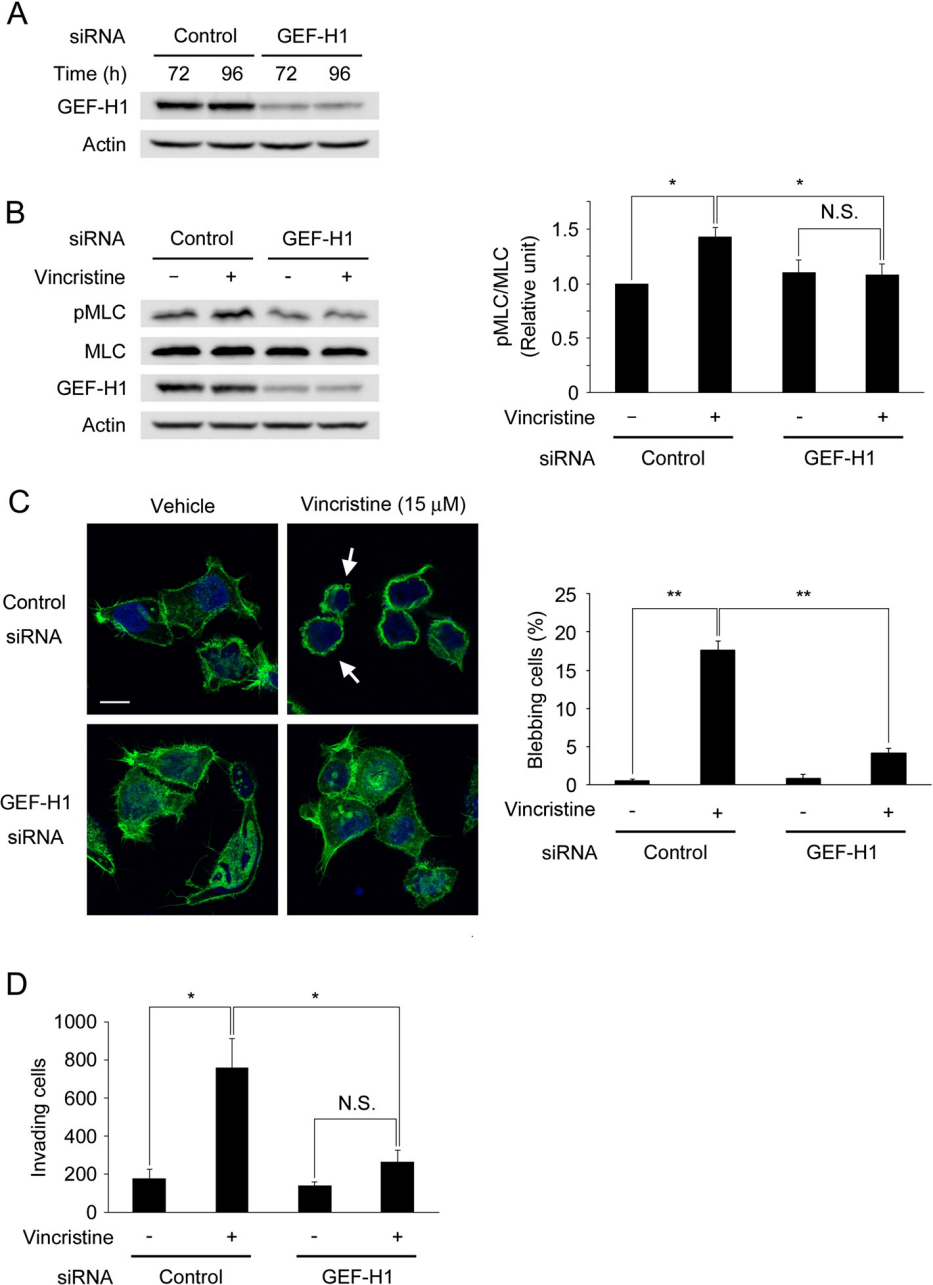
**Involvement of GEF-H1 in vincristine-enhanced invasive ability. A**. At 72 or 92 h after transfection of GEF-H1-specific siRNA or negative control siRNA, cells were harvested to analyze GEF-H1 expression by Western blotting using anti-GEF-H1 and anti-actin antibodies. Actin was used as a loading control. **B**. At 72 h after transfection, starved cells (24 h) were treated with or without 15 μM vincristine for 15 min. The cells were then lysed and analyzed by Western blotting. The blots were quantified by densitometry, and the results were expressed as a ratio relative to the values of pMLC/MLC obtained in non-treated cells. The graph shows mean ± S.E. of three independent experiments. **, *P* < 0.01. **C**. Control siRNA- or GEF-H1 siRNA-transfected cells on gelatin-coated coverslips were treated with vehicle or 15 μM vincristine. The cells were fixed and stained with Alexa Fluor 488 phalloidin and DAPI. F-actin (green) and nuclei (blue) were analyzed using confocal microscopy. Arrows indicate the cells with membrane blebs. White bars, 10 μm. The graph shows means ± S.E. of three independent experiments. **, *P* < 0.01. **D**. The number of invading cells measured by invasion assay. At 72 h after transfection, cells (1 × 10^6^) were seeded into the upper chamber with or without 15 μM vincristine. After 24 h incubation, the invading cells were fixed, and stained with toluidine blue. Total numbers of the stained cells were counted using a microscope. The graph shows means ± S.E. of three independent experiments. *, *P* < 0.05.

Switching of the inactive (GDP-bound) form of RhoA to the active (GTP-bound) form is catalyzed by at least 24 guanine nucleotide exchange factors (GEFs) [36]. GEF-H1 is one of the RhoA-specific GEFs and a microtubule-associated RhoA activator. Because GEF-H1 becomes activated when it is released from microtubules and microtubule depolymerization activates GEF-H1 [37-39], we hypothesized that GEF-H1 was a key regulator of the vincristine-induced cellular invasion in MKN45 cells. To study the involvement of GEF-H1, endogenous GEF-H1 was down-regulated by the specific siRNA and its effect was analyzed. After MKN45 cells were treated with control or GEF-H1-specific siRNA for 72 or 96 h, the expression level of GEF-H1 was evaluated by Western blotting. As shown in Figure 6A, GEF-H1 siRNA clearly reduced GEF-H1 expression. Fifteen micromolar vincristine significantly promoted MLC phosphorylation in control siRNA-transfected cells but not in GEF-H1-depleted cells (Figure 6B). When the effect of GEF-H1 siRNA on the vincristine-induced membrane blebbing was evaluated, it significantly decreased the proportion of vincristine-induced blebbing cells (Figure 6C). Furthermore, we determined whether GEF-H1 depletion affected the vincristine-induced cellular invasive ability. While 15 μM vincristine significantly enhanced invasive ability in control siRNA-transfected cells, it did not increase that in GEF-H1-depleted cells as much as in control siRNA-transfected cells (Figure 6D). These results indicated that 15 μM vincristine enhanced the membrane blebbing and the cellular invasive ability via GEF-H1/RhoA/ROCK/MLC signaling.

## Discussion

The goal of this study was to elucidate whether four different anti-cancer drugs (vincristine, paclitaxel, cisplatin and etoposide) could prompt invasive ability of tumor cells. We studied cellular invasive ability and intracellular signaling using these anti-cancer drugs in MKN45 cells, and report four main findings here. First, only vincristine, but not the other anti-cancer drugs, enhanced cellular invasive ability in MKN45 cells (Figure 1). Second, it induced the formation of membrane blebs and amoeboid-like motility (Figure 2). Third, it induced GEF-H1/RhoA/ROCK/MLC signaling (Figures 3-6). Fourth, GEF-H1 and ROCK mediated vincristine-induced cellular invasive ability (Figures 4 and 5). These results indicated that vincristine enhanced amoeboid-like motility via GEF-H1/RhoA/ROCK/MLC signaling, thereby promoting invasive ability in MKN45 cells.

Vincristine is widely used in the treatment of leukemia, lymphoma, myeloma, glioma, solid tumors of childhood often combined with other drugs. Lung, breast, and cervical cancer are also treated with vincristine. Vincristine is used at up to 2 mg/body in one administration. When 2 mg vincristine is injected i.v. to a patient, its blood concentration is estimated to be within 10–400 nM for a few hours [40]. Furthermore, it was reported that vincristine accumulated to some tissues such as spleen, thyroid, large and small intestine, and the local concentrations in these organs could reach to 6–70 times higher than that in blood [41]. These data suggest that the maximum concentration of vincristine is in the range of 0.06-28 μM in some organs of a patient treated with 2 mg vincristine. Therefore, it is conceivable that 15 μM vincristine, the maximum dose we used in this study, is physiologically-achievable.

We found that vincristine enhanced cellular invasive ability of MKN45 cells in a concentration-dependent manner. Previously, Zhao et al. have shown that the IC50 of vincristine (72 h treatment) in MKN45 cells is about 7 μM [42]. Because we observed the invasion-stimulating effect by vincristine at as low as 1 μM after 24 h treatment (Figure 1A), which is lower than IC50, we assume that this is not a non-specific effect of vincristine. However, this finding contradicts the data that microtubule depolymerizers inhibit cellular invasion observed in other studies [7-9]. The concentrations of vincristine used in our study were higher than the concentrations in the other studies [7-9]. Therefore, we consider that one potential reason for this discrepancy in the effect of vincristine on cellular invasiveness could be due to the concentration of vincristine used, and only high concentration vincristine would be able to induce GEF-H1/RhoA/ROCK/MLC signaling, leading to high cellular invasiveness. In addition, since the cell types and the assay systems to measure cellular invasion used in these studies are different from those used in this study, we cannot exclude the possibility that these factors also contributed to the outcome. To test the differences in the effect of vincristine on cellular invasion among cell types, we examined it using human lung adenocarcinoma A549 cells and human cervical adenocarcinoma HeLa cells. Whereas vincristine stimulated cellular invasive ability in A549 cells similar to MKN45 cells, it was not increased in HeLa cells (data not shown). These results suggest that the enhancement of cellular invasive ability by vincristine is at least in part cell type-specific.

In the present study, we observed no significant difference in the cell viability in 0.1 and 15 μM vincristine-treated cells (Figure 1B). This result is supported by the data reported by Warlters et al. showing that 0.1 and 11 μM vincristine exhibited the same level of cell toxicity in MKN45 cells [43]. On the other hand, we observed that the effects on invasive ability were significantly different between 0.1 and 15 μM vincristine. These results suggested that vincristine enhanced cellular invasive ability in a concentration-dependent manner without affecting the viability in MKN45 cells.

In contrast to vincristine, paclitaxel had a strong inhibitory effect on cellular invasive ability (Figure 1A). Paclitaxel has been shown to inhibit RhoA activity [44]. Because RhoA activity is required not only for amoeboid-like motility but also for general cellular motility [22,30,45,46], it is possible that paclitaxel attenuated cellular invasion by inhibiting RhoA activity. Although both vincristine and paclitaxel act on microtubules as anti-cancer drugs, our results indicate that they influence cellular motility differently depending on the effect on RhoA activity. In addition, microtubule depolymerization is shown to activate GEF-H1 [37-39]. Therefore, paclitaxel may inhibit GEF-H1 activity through the inhibition of microtubule depolymerization, thereby inhibiting the signaling pathway leading to cellular motility.

MLC phosphorylation induces actomyosin contraction, which is required for the formation of membrane blebs [17,25,27,35]. As shown in Figure 2A, 15 μM vincristine induced the formation of membrane blebs, which were not observed in control cells or in the cells treated with 0.1 μM vincristine. Consistent with this result, 15 μM vincristine induced MLC phosphorylation whereas 0.1 μM vincristine did not (Figure 3B). Therefore, we assume that the difference in the effects of vincristine on the formation of membrane blebs is attributable to MLC phosphorylation induced by GEF-H1/RhoA/ROCK signaling. As mentioned above, microtubule depolymerization activates GEF-H1 [37-39], promoting RhoA/ROCK/MLC signaling. It is thus possible that severe depolymerization of microtubules by 15 μM vincristine, but not by 0.1 μM vincristine, stimulates GEF-H1/RhoA/ROCK/MLC signaling, resulting in the formation of membrane blebs (Figure 2A).

The functions of microtubules in amoeboid-like motility are not well understood [27]. In this study, we showed that vincristine enhanced amoeboid-like motility. Because vincristine is a microtubule depolymerizer, our results may provide evidence that amoeboid-like motility does not require structural functions of microtubules. This concept will be clarified by performing further studies such as the live-cell fluorescence imaging of microtubules in vincristine-induced amoeboid-like moving cells.

To the best of our knowledge, this is the first report to provide evidence that GEF-H1 can regulate amoeboid-like motility. Previous studies have reported that GEF-H1 regulates the interaction of actin and microtubule at the leading edge and focal adhesion turnover [47,48] that are involved in mesenchymal motility [49,50], suggesting the involvement of GEF-H1 in this mode of cellular motility. Considering these findings together with the role of GEF-H1 in amoeboid-like motility that we presented in this study, it seems likely that GEF-H1 regulates not only mesenchymal motility but also amoeboid-like motility depending on the situation. In recent studies, tumor necrosis factor-β and TGF-β have been reported to promote cellular invasion and metastasis [51-54]. These cytokines have been reported to activate or up-regulate GEF-H1 [55-57]. Additionally, radiation and doxorubicin have been shown to induce metastasis and invasion of tumor cells via TGF-β, [3]. Therefore, pathophysiological conditions that increase these cytokines such as inflammation might stimulate cellular invasion via the activation and/or up-regulation of GEF-H1.

As described above, vincristine has been reported to accumulate in some organs at higher concentration than in blood after administration [41]. Given the fact that vincristine is widely used in cancer treatment, we surmise that vincristine treatment to cancer patients could adversely induce the invasion of tumor cells in some organs when its local concentration increases in the clinical setting. If this is the case, it would be beneficial to inhibit GEF-H1/RhoA/ROCK/MLC signaling pathway when treated with vincristine to prevent tumor metastasis.

## Conclusions

**Figure 7:**
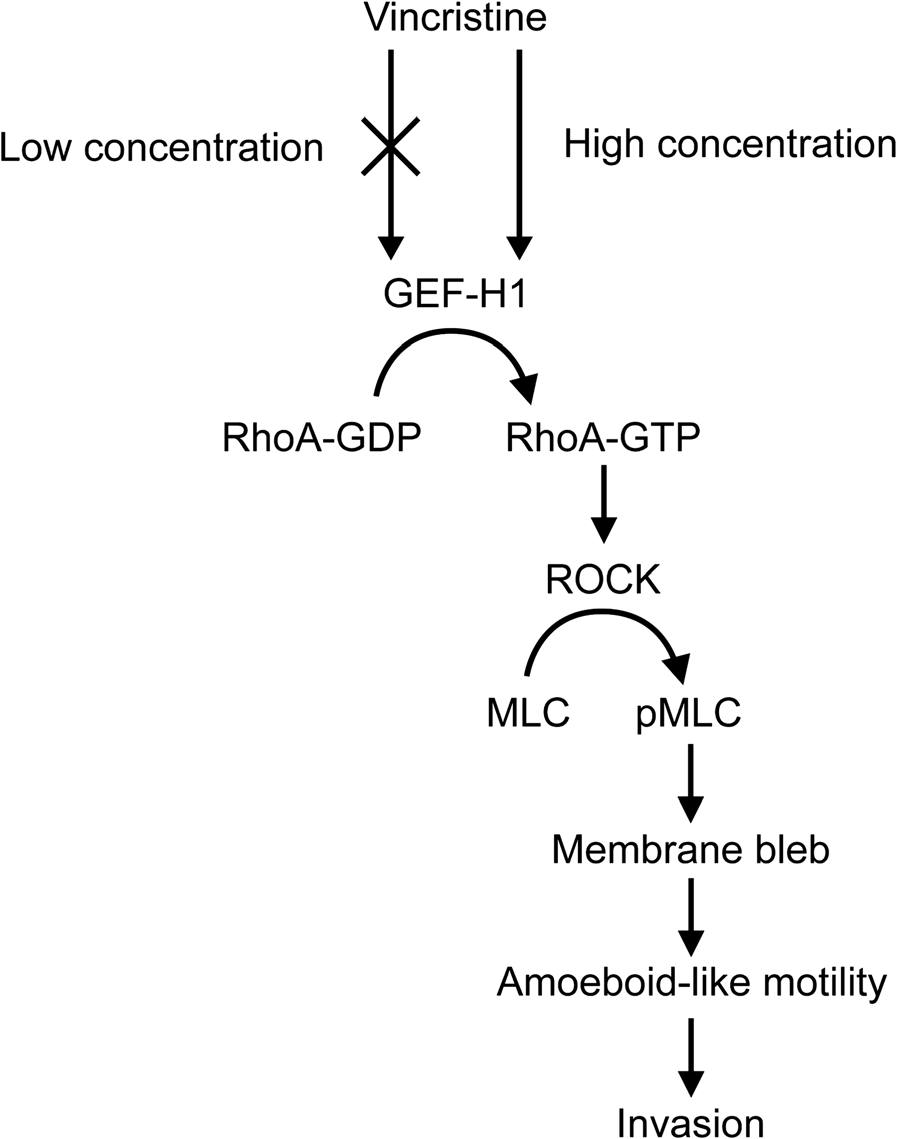
Summary of this study.

In conclusion, as summarized in Figure 7, this study indicates that vincristine enhances amoeboid-like motility via GEF-H1/RhoA/ROCK/MLC signaling in MKN45 cells. Our results provide a new insight into anti-cancer drug-induced invasion of tumor cells.

## Abbreviations

ECM: Extracellular matrix; F-actin: Filamentous-actin; FBS: Fetal bovine serum; GEF: Guanine nucleotide exchange factor; MLC: Myosin light chain; MMPs: Matrix metalloproteinases; PBS: Phosphate-buffered saline; pMLC: Phosphorylated MLC; ROCK: Rho-associated protein kinase; siRNA: Small interfering RNA; TGF-β: Transforming growth factor-β.

## Competing interests

The authors declare that they have no competing interests.

## Authors’ contributions

ME, SM and HY performed the research, analyzed the data, and prepared the manuscript. TY and OI designed the research, interpreted the data. All authors approved the final version of the manuscript.

## Funding

This work was supported, in part, by Grants-in-Aid for Basic Scientific Research from the Ministry of Education, Culture, Sports, Science and Technology, Japan (No. 21658106, No. 21380185 [O.I.] and No. 21780267 [T.Y.]), and by the Akiyama Life Science Foundation (T.Y. and H.Y.).

## Pre-publication history

The pre-publication history for this paper can be accessed here:

http://www.biomedcentral.com/1471-2407/12/469/prepub

## Supplementary Material

Additional file 1 ** Video S1.** (.wmv, 3.94 MB). Time-lapse image sequence of control MKN45 cell in 3D Matrigel environment. Click here for file

Additional file 2 ** Video S2.** (.wmv, 4.55 MB). Time-lapse image sequence of MKN45 cells treated with 15 µM vincristine in 3D Matrigel environment. Click here for file

Additional file 3 ** Video S3.** (.wmv, 5.39 MB). Time-lapse image sequence of MKN45 cells treated with 15 µM vincristine in 3D Matrigel environment. Click here for file
